# Oxygen Requirement and Associated Risk Factors in Post-COVID-19 Patients Admitted to a Tertiary Care Center: A Cross-Sectional Study

**DOI:** 10.1155/2023/3140708

**Published:** 2023-11-14

**Authors:** Bishnu Deep Pathak, Binit Upadhaya Regmi, Sushil Joshi, Bishal Dhakal, Suhail Sapkota, Kanchan Bishwakarma, Ashim Bhandari, Seejan Pathak, Shriya Sharma, Aakriti Adhikari, Nabin Simkhada, Dhan Shrestha

**Affiliations:** ^1^Nepalese Army Institute of Health Sciences, College of Medicine, Kathmandu, Nepal; ^2^Department of Internal Medicine, Nepalese Army Institute of Health Sciences, College of Medicine, Kathmandu, Nepal; ^3^Department of Internal Medicine, Mount Sinai Hospital, Chicago, Illinois, USA

## Abstract

**Background:**

COVID-19 commonly affects the lungs and may lead to mild to severe hypoxemia. The supplemental oxygen requirement gradually reduces with the improvement in lung pathology. However, a few patients may have exertional desaturation, and ongoing oxygen needs at the time of hospital discharge. The objective of this research was to study the requirement of oxygen therapy in the immediate post-COVID-19 period and its associated risk factors.

**Materials and Methods:**

An analytical cross-sectional study was conducted on the admitted post-COVID-19 patients who had recently tested real-time polymerase chain reaction (RT-PCR) negative in a tertiary care center from August 2021 to mid of October 2021. Nonprobability consecutive sampling was used, and the sample size was 108. The data were analyzed using the Statistical Package for the Social Sciences (IBM-SPSS), version 23. The mode of oxygen therapy (nasal cannula, face mask, reservoir mask, or mechanical ventilation) in the first two weeks of the study was presented appropriately in a table. The nonparametric statistical tests were applied to determine the association between the duration of post-COVID-19 oxygen therapy and several other risk factors such as age, gender, comorbidities, smoking status, exposure to firewood, COVID-19 vaccination, and severity of COVID-19.

**Results:**

95 (87.96%) cases required oxygen therapy in their immediate post-COVID-19 period. The overall median duration of oxygen therapy was 6.00 (4.00–10.00) days. The nasal cannula was the most commonly used mode of oxygen supplement. The duration of oxygen therapy was significantly higher in patients aged more than 60 years (6.00 [5.00–11.00], *p* = 0.013), chronic obstructive pulmonary disease (10.00 [6.00–12.75], *p* = 0.006), history of chronic smoking (9.00 [5.50–13.00], *p* = 0.044), and severe COVID-19 infection (7.00 [5.00–10.50], *p* = 0.042).

**Conclusions:**

The proportion of patients requiring oxygen therapy in the immediate post-COVID-19 period was higher than that reported in other studies. In addition, old age (>60 years), chronic obstructive pulmonary disease, chronic smoking, and severe COVID-19 infection significantly increased the duration of oxygen therapy. So, these factors should be assessed while discharging patients from COVID-19 facilities, and oxygen supplementation should be planned for needy patients.

## 1. Introduction

Severe acute respiratory syndrome coronavirus 2 (SARS-CoV-2) was identified as a causative agent for respiratory infection in the Hubei Province of Wuhan, China, in December 2019. It was later termed coronavirus disease 2019 (COVID-19) and was declared a pandemic by the World Health Organization (WHO) on March 11, 2020 [1]. As of 06 April 2023, more than 762 million confirmed cases and 6.89 million deaths have been reported globally [2]. The majority of the cases are mild to moderate in severity; however, 5–10% present with severe and life-threatening disease. These patients may develop respiratory distress or respiratory failure and may require intensive care. The mortality rate due to COVID-19 approximates two percent [3, 4]. The duration of symptoms is proportional to the severity of the disease [5].

The most affected organ with COVID-19 is the lungs, with a spectrum of injuries that include diffuse alveolar epithelium destruction, hyaline membrane formation, capillary damage and bleeding, alveolar septal fibrous proliferation, and pulmonary consolidation [6]. This makes hypoxemia a common entity in hospitalized COVID-19 patients. Hypoxic respiratory failure is a common complication in severe cases. Although supplemental oxygen requirements reduce over time along with improvement in underlying lung pathology, some patients will certainly have ongoing oxygen needs at hospital discharge. Additionally, in the recovery phase, patients may have desaturation on exertion [7].

Oxygen therapy is an essential component of treatment even during the recovery period. The need for this therapy is dependent upon various factors related to the patient's health. Adequate knowledge of these risk factors could possibly help clinicians to decide the best mode of management for different groups of patients. To our best knowledge, very few studies have been conducted regarding oxygen therapy in the post-COVID-19 period. Therefore, the objective of our research was to study the requirement of oxygen in immediate post-COVID-19 patients, who have just tested real-time polymerase chain reaction (RT-PCR) negative, and to find its associations with several risk factors.

## 2. Materials and Methods

### 2.1. Study Setting

The study was conducted in a tertiary care teaching hospital, in Kathmandu, Nepal, from August 2021 to mid of October 2021. During this period, the COVID-19 pandemic was at its peak in Southeast Asia including Nepal. It is a 635-bedded hospital equipped with high care and intensive healthcare facilities. With the acute surge of the pandemic, lots of structural changes were made in its organization, which led to the formation of a 100-bedded general COVID-19 ward, 30 bedded COVID-19 high care unit (HCU), and 20 bedded COVID-19 intensive care unit (ICU). Likewise, the post-COVID-19 patients who had long-term intolerable symptoms and persistently required oxygen therapy were admitted in a separate ward.

### 2.2. Study Design and Participants

This was a single-center, cross-sectional study conducted on patients who had recently tested real-time polymerase chain reaction (RT-PCR) negative and were admitted to the post-COVID-19 ward either due to the requirement of oxygen therapy or due to severe and unbearable post-COVID-19 symptoms such as palpitations, severe body ache, arrhythmia, and dizziness. Only those hemodynamically stable patients who consented to participate in the study were taken. The hemodynamically unstable cases and those who did not provide consent were excluded from the study.

### 2.3. Sampling and Sample Size

We adopted a nonprobability consecutive sampling method to select patients for this study. This method was most feasible for the researchers during the chaotic period of the COVID-19 pandemic. Therefore, all the COVID-19 patients who tested RT-PCR negative were taken consecutively according to their admission to the post-COVID-19 ward within the study period.

The minimum sample size was calculated by using Cochran's formula as follows:

(1) $$\begin{array}{c} N=\frac{Z^{2}pq}{e^{2}}\\ =\frac{\left(1.96^{2}\times 0.50\times 0.50\right)}{0.1^{2}}\\ =96.04∼96, \end{array}$$

where *N* = sample size, *Z* = 1.96 at 95% confidence interval, and *p* = 0.50 = prevalence is taken as 50%. *q* = 1–*p* = 0.50, *e* = standard error (taking 10%).

However, considering the nonresponse rate of 10%, the final sample size was approximately 108.

### 2.4. Data Collection and Study Variables

The data were collected by the researchers themselves via a semistructured questionnaire prepared with the help of a literature review and in accordance with the objectives of our study. Before starting the study, it was pretested in post-COVID-19 patients at the same site, using 10% of the estimated sample size. The required information was retrieved from patients' admission files, daily notes, and from the patients themselves. The relevant information was initially recorded in proforma containing the semistructured questionnaires prepared by all the researchers beforehand. The data collection process started from August 2021 to mid of October 2021. It corresponded to the period following the acute surge of the second wave pandemic in Nepal.

The data included sociodemographic variables such as age and gender. Baseline information related to comorbidities, types of comorbidities (if present), smoking status, prolonged exposure to firewood at home, and COVID-19 vaccination status were also noted. The smoking pack years (PY) were also calculated for those who had a past history of smoking. Likewise, the patients who had been using firewood for cooking food items at home for many years were considered to have chronic exposure to firewood smoke. The information related to the COVID-19 profile of each patient was also recorded from their previous admission files kept in the record section of the hospital. It included the severity of COVID-19 infection (as diagnosed by the treating physician), duration of RT-PCR-positive status, and High-Resolution Computerized Tomography (HRCT) score. The severity of infection in these patients was categorized as mild, moderate, severe, and critical as per the WHO COVID-19 Clinical Management Guidelines (Living Guidance 25 January 2021) [8]. We followed the local protocol for oxygen administration made by the consultant physician team at our hospital. For patients without chronic obstructive lung disease (COPD) and chronic smoking, the minimum criteria for oxygen administration were SaO2 of less than 92%. For patients with COPD or with a history of chronic smoking, oxygen was administered once saturation dropped below 88% with the target to keep between 88 and 92%. All patients were followed up till discharge from hospital or death during their hospital stay. A few of the patients taken in our study were shifted to the ICU in the middle of our study due to deterioration in their clinical status. These cases were also followed accordingly.

### 2.5. Ethical Consideration

The ethical approval was taken from the Institutional Review Committee (IRC Reg. No. 438, Ref No. 245), Nepalese Army Institute of Health Sciences (NAIHS), Kathmandu, Nepal. Before conducting the study, permission was taken from the hospital authority and the ward in charge. Only those patients who provided voluntary informed consent were enrolled in this study. The privacy and anonymity of each patient were well-maintained.

### 2.6. Data Analysis

The data were entered and analyzed using Statistical Package for the Social Sciences (IBM-SPSS), version 23. First, the proportion of post-COVID-19 patients requiring oxygen therapy was calculated. After that, the average duration of oxygen requirement and its possible risk factors were assessed. The dependent variable was the duration of oxygen therapy (in days), while the rest of the factors affecting it were independent variables. The normality of continuous data was checked by using the Shapiro–Wilk W test and histogram. Mean/standard deviation and median/interquartile range (IQR) were calculated for normally and non-normally distributed variables, respectively. The nonparametric tests were applied to assess the association between the duration of post-COVID-19 oxygen therapy with other independent variables.The tests used in the analysis were the Mann–Whitney *U* test and the Kruskal–Wallis H test for dichotomous and multichotomous variables, respectively. Likewise, the Spearman correlation was also used to assess the relationship between two continuous variables. The significance level was taken as *p* < 0.1, with a 95% confidence interval throughout the analysis.

## 3. Results

A total of 108 post-COVID-19 patients were taken. Out of these, 95 (87.96%) cases required oxygen therapy. The overall median duration of oxygen therapy was 6.00 (4.00–10.00) days, with minimum and maximum durations being two days and 30 days, respectively.

Table 1 shows the modes of oxygen administration in post-COVID-19 patients during the first two weeks of the study period. The nasal cannula was the most commonly used mode of oxygen therapy. Two patients under noninvasive mechanical ventilation expired during the period. Of these, one patient receiving continuous positive airway pressure (CPAP), and the other under bilevel positive airway pressure (BiPAP) died on the sixth and seventh days of admission, respectively.

### 3.1. Sociodemographic Characteristics

Among those who required oxygen therapy, 57 (60.00%) were males, and 38 (40.00%) were females. The mean age was 59.31 ± 14.68 years. Of these, 46 (48.42%) patients belonged to less than or equal to 60 years, and 49 (51.58%) were above 60 years of age. The age was mildly positively correlated with the duration of oxygen therapy in the post-COVID-19 period (*ρ* = 0.205, *n* = 95, *p* = 0.046). Likewise, the median duration of oxygen therapy was significantly higher in the older age group (>60 years) compared to younger ones (6.00 [5.00–11.00] vs. 5.50 [3.00–8.00], *p* = 0.013). However, there was no significant difference in terms of gender (Table 2).

### 3.2. Comorbidities

The most common comorbidities were hypertension (39, 41.1%), followed by chronic obstructive pulmonary disease (COPD) (22, 23.2%) and diabetes mellitus (19, 20.00%). In addition, the presence of COPD was significantly associated with a longer duration of oxygen therapy (*p* = 0.006). However, there was no association with other comorbidities (Table 2).

### 3.3. Smoking Status and Exposure to Firewood

**Figure 1:**
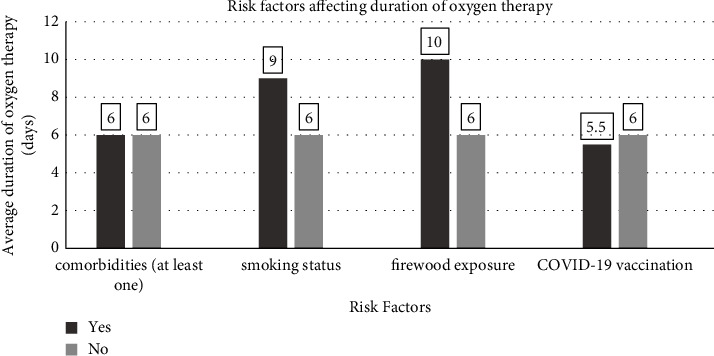
Risk factors affecting the duration of oxygen therapy in the post-COVID-19 period.

17 (17.9%) patients had a history of chronic smoking, with average pack years being 12.50 (10.00–20.00). The duration of post-COVID-19 oxygen therapy was significantly higher in them compared to nonsmokers (9.00 [5.50–13.00] vs. 6.00 [3.75–10.00], *p* = 0.044). However, there was no significant correlation between smoking pack years and the duration of oxygen therapy (*ρ* = 0.192, *n* = 17, *p* = 0.460). Likewise, five patients (5.3%) gave a history of chronic exposure to firewood. No significant association (*p* = 0.152) was found between firewood exposure and the duration of oxygen therapy in the post-COVID-19 patients (Table 2) (Figure 1).

### 3.4. COVID-19 Profile

Out of total oxygen-requiring post-COVID-19 patients, 37 (38.95%), 33 (34.74%), and 25 (26.32%) were previously diagnosed with severe, moderate, and mild COVID-19 by the treating physician. The duration of oxygen therapy was significantly higher in severe (7.00 [5.00–10.50]) compared to mild (5.00 [3.00–7.00]) and moderate cases (6.00 [4.00–11.50]) (*p* = 0.042).

The patients, who had not received COVID-19 vaccination, had a longer duration of post-COVID-19 oxygen requirement (6.00 [4.00–10.00]) compared to vaccinated ones (5.50 [3.75–15.00]). However, it was not statistically significant (*p* = 0.917) (Figure 1). The median duration of PCR-positive status was 14.00 (10.00–16.00) days. Likewise, there was no significant correlation between the duration of oxygen therapy and the duration of PCR-positive status (*ρ* = 0.063, *n* = 95, *p* = 0.544).

The high-resolution computerized tomography (HRCT) report of 41 patients was available during our study period. Among them, the median score was 17.00 (14.50–20.00). However, no significant correlation (*ρ* = 0.139, *n* = 41, *p* = 0.386) was found between the HRCT score and the duration of post-COVID-19 oxygen therapy (Table 2).

## 4. Discussion

In our study, most of the patients (87.96%) required oxygen therapy during their immediate post-COVID-19 period. This proportion seems to be very high. One of the reasons could be due to the inclusion of only hospital-admitted patients, and the leading cause of admission was breathlessness and desaturation. Hence, most of them were admitted for oxygen administration. Likewise, the overall average duration of oxygen therapy was 6.00 (4.00–10.00) days. This is in contrast with a study from India by Ray et al. [9] that showed the mean duration of oxygen administration was 4.6 ± 1.7 days in the subgroup in which complete weaning from oxygen therapy was possible. However, in the same study [9], the in-hospital oxygen requirement among the patient group in which weaning off oxygen was not possible was 6.0 ± 3.1 days. These differences could be due to the larger sample size in this study compared to ours. Moreover, different groups of patients in different healthcare settings could have brought these different findings. Another study reported that 35.1% of patients without pre-COVID-19 oxygen requirements needed oxygen therapy after discharge from the hospital [10]. Another study showed persistence of breathlessness in 42.6% and 65.6% of the post-COVID-19 patients discharged from wards and ICU, respectively [11]. This shows that the incidence of breathlessness/dyspnea and oxygen requirement in post-COVID-19 patients is variable across a few studies done till date. These significant variations could be attributed to differences in study population, sample size, sampling technique, and different healthcare settings. Moreover, different variants of the coronavirus could have brought this discrepancy.

In this study, the male/female ratio (M : F) of patients requiring oxygen was 1.5 : 1. This was comparatively lower than that reported in a study by Ray et al. (M : *F* = 2.2 : 1). This could be due to differences in the study site and selection of study sample. In the present study, the duration of post-COVID-19 oxygen therapy was positively correlated with the age of the patients. The duration was significantly higher in older age (>60 years) compared to younger age (≤60 years). This was supported by another study which depicted that elderly people (>60 years) had a higher risk for prolonged oxygen requirement [9]. Similarly, according to Stanford Medicine, COVID-19 patients above 75 years of age require domiciliary oxygen therapy for a longer time [12]. Another study also showed that the healing of lung lesions due to COVID-19 takes a longer duration in old age patients [13].

In our patients, the most common comorbidity was hypertension (41.05%), followed by COPD (23.16%). On the contrary, in a study from India [9], diabetes mellitus type II was most common. Furthermore, these patients were found to have higher oxygen requirements for a more extended period. In contrast, our study showed that COPD patients needed a significantly longer duration of oxygen therapy (10.00 [6.00–12.75]) compared to other comorbidities.

At its inception, the assumption that COVID-19 ends with the resolution of symptoms and avoidance of mortality was made. This led to the main focus on early recognition and treatment with antivirals, immune modulators, and cytokine-targeted therapies to avoid the overwhelming immune response causing multiorgan dysfunction syndrome (MODS). However, persistent physical, medical, and cognitive sequelae, including persistent immunosuppression and pulmonary, cardiac, and vascular fibrosis, were recognized following COVID-19. This was termed a persistent post-COVID-19 syndrome (PPCS), also referred to as long COVID-19 [14]. Among survivors, 57% had one or more long-term health consequences during the first six months, including shortness of breath, chest/throat pain, anosmia, ageusia, fatigue, headache, abdominal symptoms, cognitive symptoms, anxiety, and depression [15, 16].

Complete pulmonary recovery in COVID-19 does not occur immediately after viral clearance [17]. The predominant patterns of lung abnormalities are ground-glass opacities followed by fibrosis and pleural thickening. It was seen that the recovery of these lesions occurred gradually over the first three weeks. Therefore, post-COVID-19 patients may suffer from dyspnea and shortness of breath from minimal physical activities [18, 19]. The studies have shown that pulmonary rehabilitation is useful for the survivors of COVID-19. It has been found to decrease the length of hospital stay and improve respiratory functions. One of the important components of pulmonary rehabilitation is oxygen therapy [20].

A study from Pakistan reported that morbidity and mortality were highest among elderly patients, which is in line with our findings. In this study, 14.4% of patients who were initially admitted with the diagnosis of COVID-19 needed constant observation in the hospital compared to others. Out of these, 17.86% eventually died despite of good care and support. It signifies that some patients may take a longer time for complete recovery and health maintenance. However, their life is still in danger till complete recovery [21].

Our study has a few limitations to be mentioned. It is a single-center study with a small sample size. The post-COVID-19 patients admitted to the isolation ward have been taken for the study. Thus, the findings may not be generalisable. Moreover, a few of our cases still had an exertional desaturation at discharge. They were discharged home with oxygen cylinders, and a nasal cannula to be used at the time of need. We could not follow up on these patients. This could bring bias on the average duration of oxygen therapy recorded for these cases.

Even though our study has many limitations, it still has many implications. This study is the first of its kind conducted in post-COVID-19 patients in our setting. It sheds light on the need for oxygen therapy and its affecting factors in the immediate post-COVID-19 period. It emphasizes that virological clearance is not only the complete recovery from COVID-19 but also the patients need to be taken care of for several weeks after that. It highlights the need for oxygen administration in many such patients for a variable period. Moreover, our study illustrated some of the risk factors that could significantly affect the duration of oxygen therapy in the post-COVID-19 period. This information would be valuable for the clinicians at the time of discharge of patients from COVID-19 facilities. This could guide on management of existing risk factors that could affect the health of post-COVID-19 patients. Moreover, this study encourages further studies with improved research design and higher sample sizes to be conducted in the future so as to explore additional knowledge on this topic.

## 5. Conclusions

The proportion of post-COVID-19 patients requiring oxygen was higher compared to other studies. In addition, older age (>60 years), presence of COPD, history of chronic smoking, and severe COVID-19 infection were significantly associated with a longer duration of oxygen therapy during the immediate post-COVID-19 period. So, these factors should be assessed appropriately while discharging patients from COVID-19 facilities, and oxygen supplementation should be planned for needy patients.

## Figures and Tables

**Table 1:** Modes of oxygen therapy in post-COVID-19 patients during the first two weeks of study.

Day of admission	Total patients (*n*)	Nasal cannula *n* (%)	Face mask *n* (%)	Reservoir mask *n* (%)	CPAP *n*(%)	BiPAP *n*(%)
1st	95	64 (67.37)	21 (22.11)	8 (8.42)	1 (1.05)	1 (1.05)
2nd	89	62 (69.66)	17 (19.10)	8 (8.99)	1 (1.12)	1 (1.12)
3rd	73	53 (72.60)	13 (17.81)	5 (6.85)	1 (1.37)	1 (1.37)
4th	58	44 (75.86)	9 (15.52)	3 (5.17)	1 (1.72)	1 (1.72)
5th	50	38 (76.00)	7 (14.00)	3 (6.00)	1 (2.00)	1 (2.00)
6th	40	31 (77.50)	5 (12.50)	2 (5.00)	1 (2.50)	1 (2.50)
7th	31	23 (74.19)	4 (12.90)	1 (3.23)	0	3 (9.68)
8th	30	26 (86.67)	0	2 (6.67)	0	2 (6.67)
9th	25	21 (84.00)	0	2 (8.00)	0	2 (8.00)
10th	25	20 (80.00)	0	3 (12.00)	0	2 (8.00)
11th	17	11 (64.71)	2 (11.76)	4 (23.53)	0	0
12th	15	9 (60.00)	4 (26.67)	2 (13.33)	0	0
13th	13	8 (61.54)	5 (38.46)	0	0	0
14th	12	11 (91.67)	1 (8.33)	0	0	0

*Note.* CPAP: continuous positive airway pressure. BiPAP: bilevel positive airway pressure.

**Table 2:** Risk factors affecting the duration of oxygen therapy in the post-COVID-19 period.

S. no.	Variables	Duration of oxygen therapy (in days)	*p* value
1	Age category		**0.013**
	Less than and equal to 60 years (*n* = 46, 48.42%)	5.50 (3.00–8.00)	
	More than 60 years (*n* = 49, 51.58%)	6.00 (5.00–11.00)	

2	Gender		0.963
	Males (*n* = 57, 60.00%)	6.00 (4.00–10.00)	
	Females (*n* = 38, 40.00%)	6.00 (3.75–10.00)	

3	Comorbidities (at least one)		0.142
	Yes (*n* = 57, 60.00%)	6.00 (4.00–10.50)	
	No (*n* = 38, 40.00%)	6.00 (3.00–8.25)	

4	Comorbidities		
	Hypertension (*n* = 39, 41.05%)	6.00 (4.00–10.00)	0.879
	Diabetes mellitus (*n* = 19, 20.00%)	5.00 (4.00–10.00)	0.594
	COPD (*n* = 22, 23.16%)	10.00 (6.00–12.75)	**0.006**
	CKD (*n* = 5, 5.26%)	6.00 (4.50–8.00)	0.828
	CVD (other than HTN) (*n* = 12, 12.63%)	6.00 (3.25–10.25)	0.844
	Hypothyroidism (*n* = 6, 6.32%)	9.50 (6.25–12.00)	0.146
	Others (*n* = 15, 15.79%)	7.00 (5.00–17.00)	**0.063**

5	Smoking status		**0.044**
	Yes (*n* = 17, 17.9%)	9.00 (5.50–13.00)	
	No (*n* = 78, 82.1%)	6.00 (3.75–10.00)	

6	Exposure to firewood		0.152
	Yes (*n* = 5, 5.3%)	10.00 (7.50–10.00)	
	No (*n* = 90, 94.7%)	6.00 (4.00–10.00)	

7	Severity of COVID-19		**0.042**
	Mild (*n* = 25, 26.32%)	5.00 (3.00–7.00)	
	Moderate (*n* = 33, 34.74%)	6.00 (4.00–11.50)	
	Severe (*n* = 37, 38.95%)	7.00 (5.00–10.50)	

8	COVID-19 vaccination status		0.917
	Yes (*n* = 10, 10.53%)	5.50 (3.75–15.00)	
	No (*n* = 85, 89.47%)	6.00 (4.00–10.00)	

*Note.* Duration of oxygen therapy (in days) expressed as median (interquartile range = *Q*1 – Q3). The *p* value is derived from the Mann–Whitney *U* test and Kruskal–Wallis H test for dichotomous and multichotomous variables, respectively. All bold *p* values are statistically significant (*p* < 0.1). Other comorbidities include psychiatric disorders, autoimmune disorders, dyslipidemia, and stroke. COPD: chronic obstructive pulmonary disease. CKD: chronic kidney disease. CVD: cardiovascular diseases. HTN: hypertension. COVID-19: coronavirus disease 2019.

## Data Availability

The data used for the current study are available from the corresponding author upon reasonable request.

## Additional Points

*Supporting Information*. S1: Dataset. Oxygen requirement and associated risk factors in post-COVID-19 patients (spss) https://figshare.com/s/7514109630cd4f9002d1. S2: Questionnaires.
